# Temporal variability in zooplankton community in the western Yellow Sea and its possible links to green tides

**DOI:** 10.7717/peerj.6641

**Published:** 2019-04-08

**Authors:** Weicheng Wang, Guangtao Zhang, Xiaoxia Sun, Fang Zhang, Xing Zhang

**Affiliations:** 1 Key Laboratory of Marine Ecology and Environmental Sciences, Institute of Oceanology, Chinese Academy of Sciences, Qingdao, China; 2 Jiaozhou Bay Marine Ecosystem Research Station, Institute of Oceanology, Chinese Academy of Sciences, Qingdao, China; 3 University of Chinese Academy of Sciences, Beijing, China; 4 Laboratory for Marine Ecology and Environmental Science, Qingdao National Laboratory for Marine Science and Technology, Qingdao, China; 5 Center for Ocean Mega-Science, Chinese Academy of Sciences, Qingdao, China; 6 Huangdao Sub-administration of Qingdao Meteorological Administration, Qingdao, China

**Keywords:** Community structure, Copepods, Macro-algal blooms, Size structure, *Ulva prolifera*, Yellow Sea

## Abstract

Large-scale macro-algal blooms of *Ulva prolifera* (also called green tides) have appeared each summer since 2008 in the western Yellow Sea. In this study, we investigated the temporal variability in zooplankton community in the western Yellow Sea and its possible links to green tides using data from a long-term plankton survey off the coast of Qingdao, China. Environmental conditions observed in the study area during the green tide period (GTP: June–August, 2008–2013) were compared to the non-green tide period (NGTP: June–August, 2005–2007), to support the contention that variations observed in zooplankton community may be attributed to the green tides, as opposed to natural climatic or environmental variations. Zooplankton assemblage structure observed during the GTP was then compared to the NGTP. Significant variations were detected both in zooplankton abundance and assemblage structure between the two defined periods. The abundance of zooplankton, mainly copepods, was significantly decreased during the GTP. Meanwhile, the relative abundance of copepods decreased by approximately 10% and that of gelatinous zooplankton, including appendicularians, chaetognaths, and medusae, almost doubled (ca. increased by 6.4%). The dominant species of meroplankton completely changed, specifically, polychaeta, and echinoderm larvae were more dominant than decapod and bivalve larvae. With regard to zooplankton size structure, the NGTP showed a higher size diversity with more small-sized organisms, while the GTP showed a lower size diversity in the community. According to general linear models, the interannual variation in summer zooplankton abundance was significantly correlated with green tides. These results indicate that the temporal changes in zooplankton community may have a close link to the green tides.

## Introduction

In the last decades, coastal marine ecosystems are under increasing pressure from multiple drivers related to human-induced environmental changes, including resource extraction, habitat modification and destruction, and inputs of pollutants and nutrients (Halpern et al., 2008). The increased frequency of occurrence, intensity and geographical range of ephemeral macro-algal blooms is a widespread symptom of chronic eutrophication in coastal waters (Schramm, 1999; Jones & Pinn, 2006; Zhou et al., 2015). In the recent decade, the Yellow Sea has experienced the world’s largest green tide (Liu et al., 2013; Wang et al., 2015). In 2007, a small scale green tide was observed for the first time in the center of the Yellow Sea (Sun et al., 2008). The green tide began to attract attention in 2008 due to its presence in the Olympic Sailing Course in Qingdao. The algal bloom had a severe impact on the coastal landscape and marine environment. It cost almost 100 million USD to clean-up the algae offshore of Qingdao in summer 2008 to achieve Olympic Games standards (Wang et al., 2009). Moreover, the green tide caused direct aquaculture losses of 116 million USD along the green-tide-affected coastlines (Ye et al., 2011). Since then, green tides have occurred annually in the western Yellow Sea, especially in coastal waters off Qingdao city (northern China), during the period from late spring to summer, causing serious ecological and economic problems and has attracted substantial scientific study (Hu, Hu & He, 2017).

Numerous studies on the green tides in the Yellow Sea have mainly focused on tracking the floating mats using remote sensing and numeric modeling (Liu et al., 2009; Keesing et al., 2011; Qi et al., 2016), species identification (Ye et al., 2008; Liu et al., 2010), and their growth cycles and seasonality (Hu et al., 2010; Wang et al., 2015). It is generally accepted that the causative species are mainly dominated by *Ulva prolifera* (Duan et al., 2011; Zhao et al., 2012), and its bloom originated from the Subei shoal in Jiangsu coast (180 km south of Qingdao) and drifted to the southern coast of the Shandong Peninsula, driven by a series of physical processes (Liu et al., 2009; Liu, Wang & Zhang, 2016). The coastal Yellow Sea is recognized as an important productive fishing ground as well as a spawning and nursery area for local fish and shrimp populations (Jin, Zhang & Xue, 2010; Sun et al., 2011a). However, the long-term ecological impact of the annual large-scale macro-algal bloom on Yellow Sea ecosystem is still unclear.

Zooplankton is a crucial component of marine ecosystems due to its pivotal role in marine trophic food webs (Sherr & Sherr, 2009) and its impact on biogeochemical cycling (Dam, Roman & Youngbluth, 1995). As zooplankton is mainly composed of ectotherms with short life cycles this allows for a fast response to stressors through phenotypic plasticity or evolutionary adaptation, they are able to respond rapidly to any ecosystem variability and are thus considered useful sentinel organisms (Beaugrand, 2005; Hays, Richardson & Robinson, 2005; Dam, 2013). Long-term plankton time-series play a requisite role in detecting such variability, since they are suitable tools in capturing the modes of population, the community structure and the changes at different temporal scales (Ouba, Abboud-Abi Saab & Stemmann, 2016). Given that macro-algal mats of *U. prolifera* have become increasingly common in the western Yellow Sea coastal regions in recent years (Qi et al., 2016), information on zooplankton response to the green tide is critical for estimating the impact of the green tide on coastal ecosystems in the western Yellow Sea. However, there have been no publications regarding the potential effects of green tide on zooplankton community in this area.

The goal of this study was to examine the temporal variation in zooplankton community and its possible links to large-scale *U. prolifera* blooms, based on data from a long-term plankton survey conducted within the impact region. Specifically, the aims of the present work are (1) to provide an overview on the temporal variability of zooplankton abundance and community structure and (2) to identify the possible links between zooplankton community variability and green tides that occurred between 2008 and 2013. This is the first report on temporal variability of zooplankton community and its possible links to green tides in the western Yellow Sea. The results will help identify potential threats posed by large-scale *U. prolifera* blooms to the coastal ecosystem.

## Materials and Methods

### Study area and field collections

**Figure 1:**
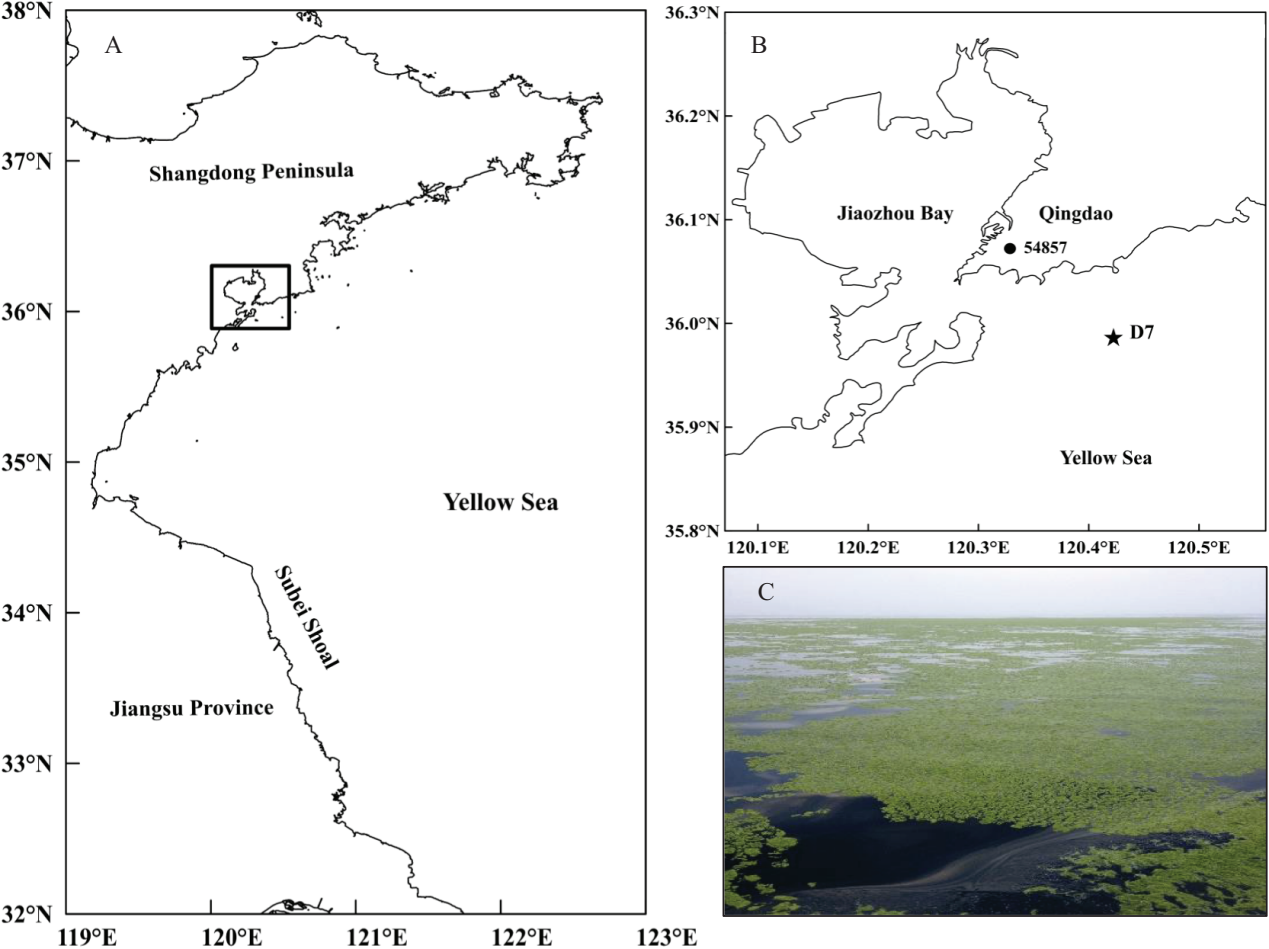
Location of the study site. (A) Map of the study area in the black rectangle and (B) the sampling station (D7) off the coast of Qingdao, northwestern Yellow Sea, and the meteorological station (54857) in Qingdao city. (C) A photo of the green tide off the coast of Qingdao was taken by Weicheng Wang on the “KE XUE SAN HAO” research vessel on June 11, 2014.

The current study was conducted at station D7 (35°59′00″N, 120°25′30″E) in the southern Yellow Sea (Fig. 1). It is located offshore of Qingdao city (northern China) with a maximum depth of approximately 18 m. This station is part of a long-term research program carried out in this area by the Jiaozhou Bay Marine Ecosystem Research Station. Since the large-scale *U. prolifera* bloom was identified in the Qingdao coast in 2008, station D7 has been continually impacted by these macro-algal blooms for a decade.

The zooplankton samples used in this study were collected by vertical tows using a conical plankton net (mesh size = 160 μm, mouth aperture = 0.08 m^2^) from near the bottom to the surface every mid-month during daytime hours from 2005 to 2013 at station D7. The net samples were immediately preserved in 5% neutral formaldehyde seawater solution. Generally, our sampling date in June each year was later than the green tide’s arrival time, except that in June 2011 due to the late arriving of green tide (Wu et al., 2014), thus we divided the month in 2011 into the non-green tide period (NGTP).

### ZooScan analysis

In the present study, the preserved zooplankton samples were analyzed and digitized with ZooScan (http://www.zooscan.com), an imaging system developed in the Laboratory of Oceanography of Villefranche (Gorsky et al., 2010). In the laboratory, a fraction of each plankton sample was collected with a Motoda box splitter (Motoda, 1959) to yield an average of ~1,000 objects per scan, in order to permit easy manual separation of organisms (Gorsky et al., 2010). Subsamples comprising 1/4–1/64 of the original samples were scanned in the scanning cell with a transparent frame (11 × 24 cm) and digitized at 4,800 dpi resolution (each pixel was equivalent to 5.29 μm^2^), according to the scanning protocol (Schultes & Lopes, 2009). Automatic recognition via supervised-learning was performed with the Plankton Identifier software. All zooplankton images were automatically classified into 11 taxonomic groups: Copepods, Appendicularians, Chaetognaths, Cladocerans, Medusae, Nauplii, Polychaete larvae, Echinoderm larvae, Decapod larvae, Bivalve larvae, and Fish eggs. The automatic classification was then manually validated to ensure accurate identification of these zooplankton groups. The images were analyzed using the dedicated imaging program Zooprocess, and the details of this process were described previously (Grosjean et al., 2004). The ZooScan digital imaging system can provide zooplankton abundance and body size, such as the equivalent spherical diameter (ESD), that is, a two-dimensional estimate of size for each organism found and classified in the samples. By default, only objects having an ESD of >300 μm were detected and processed (Grosjean et al., 2004; Gorsky et al., 2010). The abundance (ind.m^−3^) of zooplankton was calculated as follows: abundance = number of organisms in the same taxonomic group ∗ splitting ratio/net volume (Dai et al., 2016).

### Environmental data

A suite of climatic indices and environmental variables were used to determine whether variations in zooplankton community may have occurred in response to natural environmental and climatic sources of variation. A total of 10 environmental factors were collected from the Jiaozhou Bay Marine Ecosystem Research Station (http://jzw.qdio.cas.cn/), Qingdao Meteorological Administration (http://qdqx.qingdao.gov.cn/), the National Oceanic and Atmospheric Administration (http://www.noaa.gov/), and Joint Institute for the Study of the Atmosphere and Ocean (https://jisao.uw.edu/). These variables described both large-scale climatic conditions that is, pacific decadal oscillation (PDO), arctic oscillation (AO), and the East Asian summer monsoon index (EASMI), local weather and water column parameters, that is, wind speed, atmospheric pressure, rain, water temperature, and salinity. Large-scale climatic and local weather data were provided at daily intervals. Other data on water column conditions were collected at monthly intervals. All environmental data were expressed as monthly means for analyses.

### Data analysis

Principal component analyses (PCA) of quantitative environmental data were conducted to portray the temporal patterns of the environmental characteristics in the studied site. This allowed for a visual assessment of environmental conditions during the green tide period (GTP) relative to the range of historical NGTP. Convex hulls were used to group observations by months, and the relative position of group centroids was used to assess if and how GTP conditions differed from historical conditions (the NGTP).

In order to define year groupings, cluster analysis was carried out using the Bray–Curtis similarity index (Bray & Curtis, 1957) and square root data transformation. For grouping the years, the similarity indices were coupled with hierarchical agglomerative clustering with an average linkage method unweighted pair group method using arithmetic mean (Field, Clarke & Warwick, 1982). Non-metric multidimensional scaling ordination was applied to distinguish the two periods compared: the NGTP and GTP based on the square root transformed summer zooplankton taxon abundance data. The Wilcoxon rank sum test (*W*) was used to determine the differences in zooplankton abundances and community indicators between the two defined periods. The analysis of similarity (ANOSIM) routine was used to investigate differences in the relative composition of zooplankton assemblages between the NGTP and GTP by months. The contribution of taxa to community dissimilarity was calculated using similarity percentage (SIMPER) analysis.

According to ESD measures, zooplankton was divided into five size classes, that is, 0.3–0.5, 0.5–1.0, 1.0–2.0, 2.0–4.0, and >4.0 mm. Moreover, abundance data between 0.3 and 5.0 mm ESD, covering the size range of major zooplankton, were binned into 48 size classes at 0.1-mm ESD intervals. We calculated the Shannon–Weaver diversity index (Shannon & Weaver, 1949) used in the following equation for size diversity (*H′*).

$$H' = - \sum\limits_i^s {{p_i}\log _2^{{p_i}}}$$

Where *H′* = size diversity, *p_i_* = the proportion of each *i* size class and *s* = total number of size classes.

To model the effects of multiple factors (physical, meteorological, and green tide) on zooplankton abundance, a general linear model (GLM) was developed. In order to identify the influence of the green tide on the zooplankton community, we defined the values of the green tide as 0 and 1, specifically, the values of the summer months (June–August) during the NGTP were 0, and those during the GTP were 1. The adjusted coefficient of determination (*R*^2^) and Akaike information criterion (AIC) (Akaike, 1974) were used as model quality indicators to best predict the phenomena. The univariate GLMs were performed using a forward stepwise procedure, and the best-approximating GLM model with the highest *R*^2^ in combination with the lowest AIC obtained for each combination of studied factors was selected as the final model.

Analyses were performed using a combination of PRIMER 5.0 (Clarke & Warwick, 2001) and the R computing environment (R Core Team, 2014), and the significance level for all the tests was set to *P* = 0.05.

## Results

### Environmental conditions

**Figure 2:**
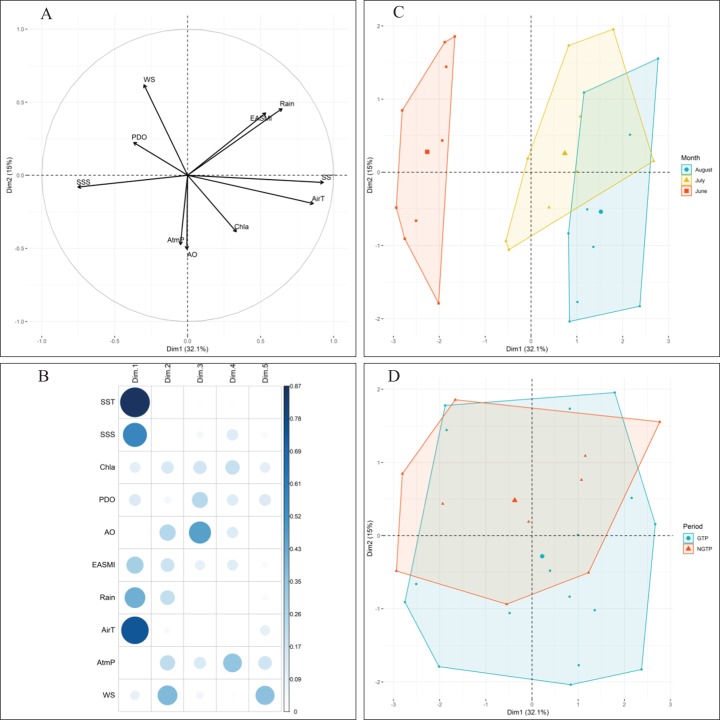
Principal component analysis (PCA) of climatic and environmental factors. With (A) correlations between variables, (B) correlations between variables and axes, and the projection of (C) monthly samples, and (D) different periods (e.g., the NGTP and green tide period GTP) on the two first principal components (Dim1-Dim2), data from 2005 to 2013 were pooled together. Data centroids (larger points) were also displayed in (C) and (D). SST, sea surface temperature; SSS, sea surface salinity; EASMI, East Asian Summer Monsoon Index; AirT, air temperature; Chl *a*, chlorophyll *a*; PDO, Pacific Decadal Oscillation; AO, Arctic Oscillation; WS, wind speed; AtmP, atmospheric pressure.

Approximately 47% of the variability in environmental conditions during June, July, and August 2005–2013 was explained by the two first components of the PCA (Fig. 2). According to the data for these 9 years, observations in June were generally characterized by strong PDO and high salinity (Fig. 2C). Conversely, water temperature, air temperature (AirT), and chlorophyll *a* (Chl *a*) were dominant, and both PDO and sea surface salinity were low in August. July was characterized by intermediate conditions. When project the monthly environmental variables between the green tide years (2008–2013) and non-green tide years (2005–2007), highly overlapping was observed (Fig. 2D). In addition, observations from the green tide years fell within the range of historical non-green tide values, as monthly centroids positioned within the convex hulls formed by historical values each month (Fig. 2D). Overall, regional environmental conditions during the green tide years were very similar to those in previous non-green tide years.

### Zooplankton abundance and taxonomic composition

**Figure 3:**
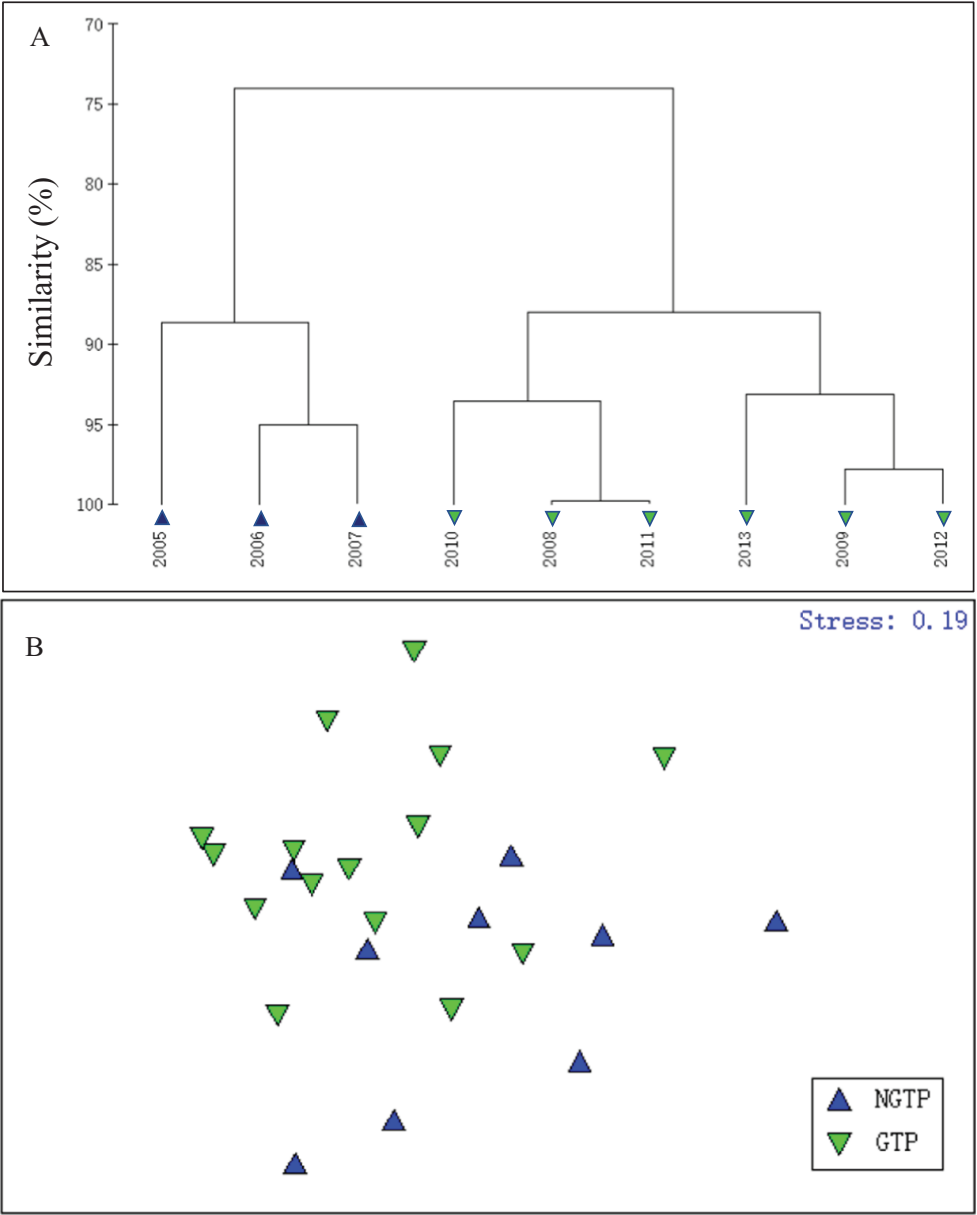
Hierarchical clustering and non-metric multidimensional scaling (NMDS) plots. (A) Dendrogram of hierarchical clustering of samples from 2005 to 2013 when considering summer total zooplankton abundance (ind. m^−3^) at the sampling station, (B) a NMDS plot on the first two axes was based on the Bray–Curtis similarities of the square root transformed zooplankton taxon abundance data for the two periods compared: the non-green tide period (NGTP) and the green tide period (GTP).

In order to define the potential effects of the green tide on zooplankton, summer total zooplankton abundances were used and cluster analysis was carried out using the Bray–Curtis similarity index and square root data transformation (Fig. 3A). According to hierarchical cluster analysis, the years studied were divided into two periods (2005–2007 and 2008–2013), which matched the large-scale appearance of the green tides off the coast of Qingdao. In addition, based on the taxon abundance, summer (June–August) zooplankton communities were classified into two groups (Fig. 3B). Thus, we divided our study years into two periods: the NGTP: June–August, 2005–2007 and the GTP: June–August, 2008–2013.

A total of 11 zooplankton taxa of different taxonomic levels were identified at the sampling station (Tables 1 and 2). ANOSIM confined significant variations in zooplankton assemblage composition during the green tide years as compared to non-green tide years. Zooplankton assemblage was different during the GTP at the sampling station when all months were combined together (*R* = 0.227, *P* < 0.05, Table 1). Assemblage significantly different from the NGTP in June (*R* = 0.256, *P* < 0.05, Table 1), but was not diverged in July and August. According to SIMPER, when all months were combined together, copepods, nauplii, and appendicularians contributed the most to differences between the two defined periods. When months were analyzed separately, copepods, chaetognaths, nauplii, bivalve larvae, medusae, appendicularians, fish eggs, decapod larvae, and echinoderm larvae contributed more than 90% of the differences between the two defined period communities (Table 1).

**Table 1:** Analyses of similarity (ANOSIM) and the contribution of dissimilarity (SIMPER).

Month	ANOSIM		SIMPER	
	*R*	*P*	Taxa	Contribution (%)
All combined	0.227	**0.013**	Copepods	15.96
			Nauplii	10.99
			Appendicularians	9.14
			Medusae	9.05
			Decapod larvae	8.85
			Polychaeta larvae	8.60
			Chaetognaths	8.14
			Bivalve larvae	8.07
			Fish eggs	7.41
			Echinoderm larvae	7.25
June	0.256	**0.024**	Copepods	26.46
			Chaetognaths	13.49
			Nauplii	12.89
			Bivalve larvae	9.43
			Medusae	7.57
			Appendicularians	6.73
			Fish eggs	6.45
			Decapod larvae	6.30
			Echinoderm larvae	4.73
July	0.031	0.393		
August	0.012	0.440		

**Note:**

ANOSIM comparing the composition of zooplankton assemblages observed from June to August between green tide years 2008–2013 and non-green tide years 2005–2007. When significant differences (*P* < 0.05) were detected, the list of taxa among the top 90% contribution of the dissimilarity (SIMPER), are listed. Bold values correspond to significant *P*-values at 0.05.

**Table 2:** Descriptive statistics of the identified zooplankton in the sampling station and the Wilcoxon test showing the difference between the non-green tide period (NGTP) and the green tide period (GTP) during summer (June–August).

Category	NGTP Average ± SE (ind. m^−3^)	GTP Average ± SE (ind. m^−3^)	*P*-value
Total zooplankton	8,781.3 ± 2,148.8	2,853.9 ± 510.2	**0.027**
Copepods	7,786.9 ± 2,033.6	2,249.5 ± 408.0	**0.029**
Nauplii	106.6 ± 30.6	23.8 ± 12.0	**0.010**
Fish eggs	38.9 ± 14.7	12.6 ± 6.9	0.185
Appendicularians	221.1 ± 74.1	160.5 ± 67.3	0.202
Chaetognaths	249.9 ± 68.2	157.9 ± 52.0	0.224
Medusae	71.7 ± 28.9	39.7 ± 21.0	0.251
Cladocerans	55.4 ± 45.7	63.9 ± 43.5	0.589
Polychaeta larvae	62.9 ± 27.6	56.5 ± 20.2	0.612
Bivalve larvae	88.7 ± 55.4	13.6 ± 6.1	0.439
Echinoderm larvae	20.6 ± 10.1	40.3 ± 17.1	0.977
Decapod larvae	78.6 ± 26.2	35.7 ± 12.5	0.198

**Note:**

Bold values correspond to significant *P*-values at 0.05.

To evaluate the effect of green tide vs non-green tide on the summer zooplankton abundance, data were pooled and then separated between the periods. Descriptive statistics (mean and standard error) of taxa abundances for each period are shown in Table 2. The difference in abundances between the two periods was significant. The test results confirmed a significant (*P* < 0.05) decrease in abundances during the GTP for total zooplankton, total copepods, and nauplii. A decrease, although not significant, in abundances was observed for fish eggs, appendicularians, chaetognaths, medusae, bivalve larvae, and decapod larvae during the GTP. Only echinoderm larvae abundance showed a substantial increase during the GTP.

**Figure 4:**
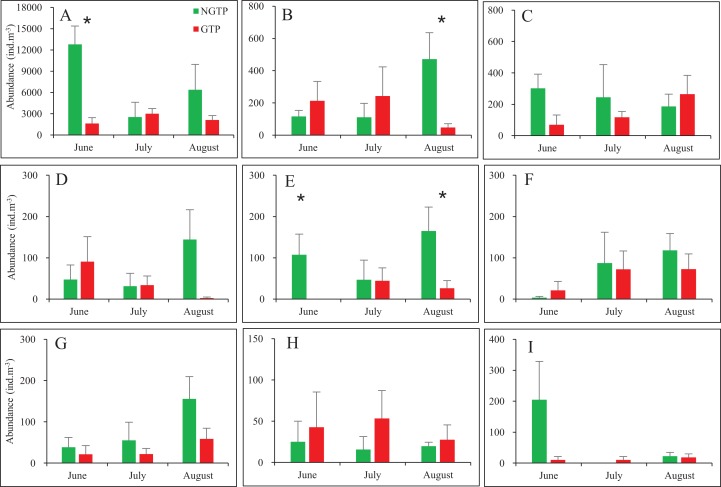
Monthly mean abundances of dominant holoplankton and meroplankton during the non-green tide period (NGTP) and green tide period (GTP) at the sampling station. (A) Copepods, (B) appendicularians, (C) chaetognaths, (D) medusae, (E) nauplii, (F) polychaeta larvae, (G) decapod larvae, (H) echinoderm larvae, and (I) bivalve larvae. Vertical bars show SE. Asterisks indicate significant differences of zooplankton taxa abundance between the NGTP and GTP.

When months were considered separately, most significant differences in abundances of taxa were observed in June and August (Fig. 4). Zooplankton found in lower abundances during the GTP including copepods (June), appendicularians (August), nauplii (June and August). No significant differences were found during any months for fish eggs, chaetognaths, medusae, cladocerans, polychaeta larvae, bivalve larvae, decapod larvae, and echinoderm larvae.

**Figure 5:**
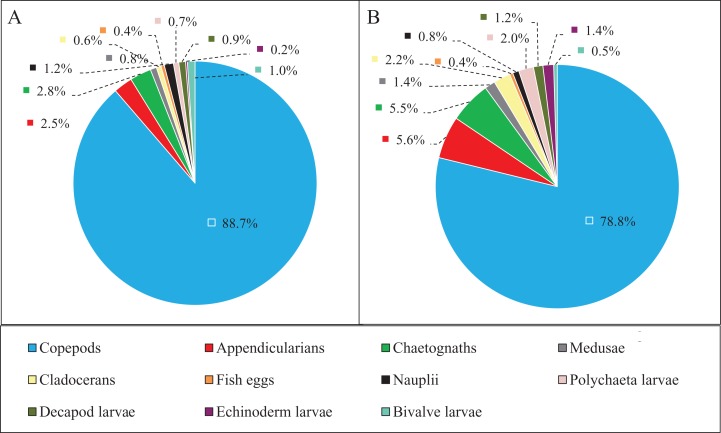
Abundance composition of zooplankton taxonomic groups. (A) The non-green tide period (NGTP) and (B) green tide period (GTP).

Zooplankton composition during each period is shown in Fig. 5. In the western Yellow Sea coastal waters, copepods were the dominant group among the enumerated organisms (88.7%), followed by chaetognaths (2.8%), and appendicularians (2.5%) during the NGTP. The difference in relative abundance between the two periods was significant. A decrease in the relative abundances of total copepods (10%), nauplii (0.4%), and bivalve larvae (0.5%) during the GTP was observed. Inversely, cladocerans, polychaeta larvae, and echinoderm larvae markedly increased (at least tripled), and medusae, chaetognaths, and appendicularians almost doubled.

**Figure 6:**
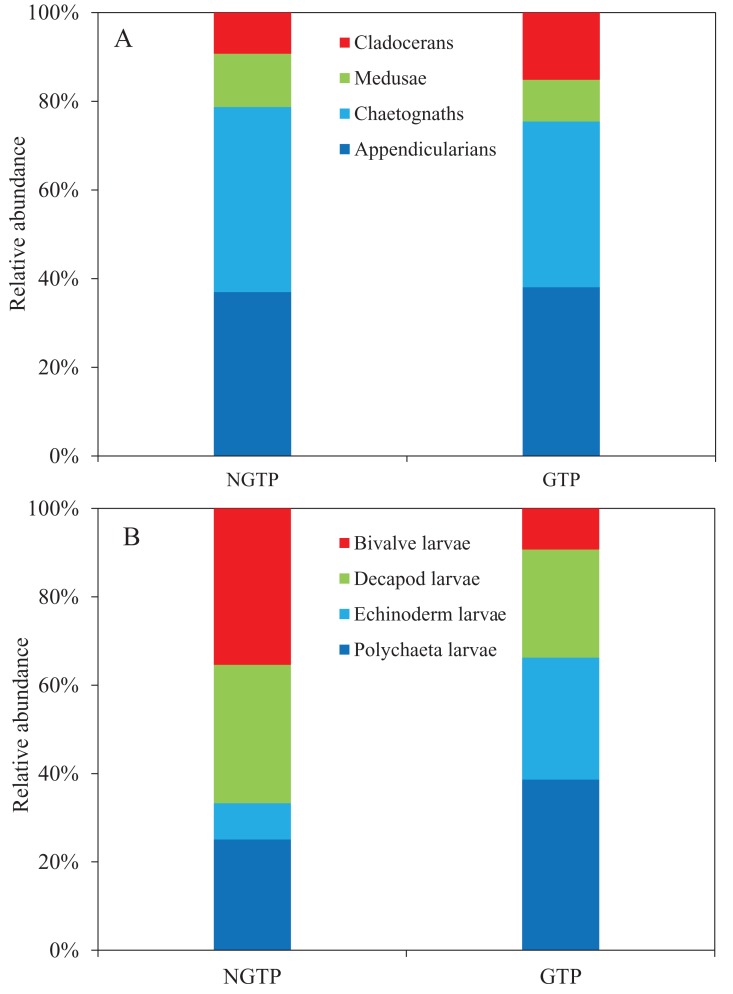
Relative abundance of zooplankton during the non-green tide period (NGTP) and green tide period (GTP) at the sampling station. (A) Dominant non-copepod holoplankton and (B) meroplankton.

Among non-copepod holoplankton (Fig. 6A), chaetognaths were dominant and cladocerans were least dominant during the NGTP. However, appendicularians increased and were the dominant group, and medusae were the least dominant group during the GTP. Similarly, the structure of meroplankton also changed (Fig. 6B). During the NGTP, bivalve larvae were dominant, followed by decapod larvae, polychaeta larvae, and echinoderm larvae, whereas, during the GTP the order of dominance was polychaeta larvae, echinoderm larvae, decapod larvae, and bivalve larvae.

### Zooplankton size structure

**Figure 7:**
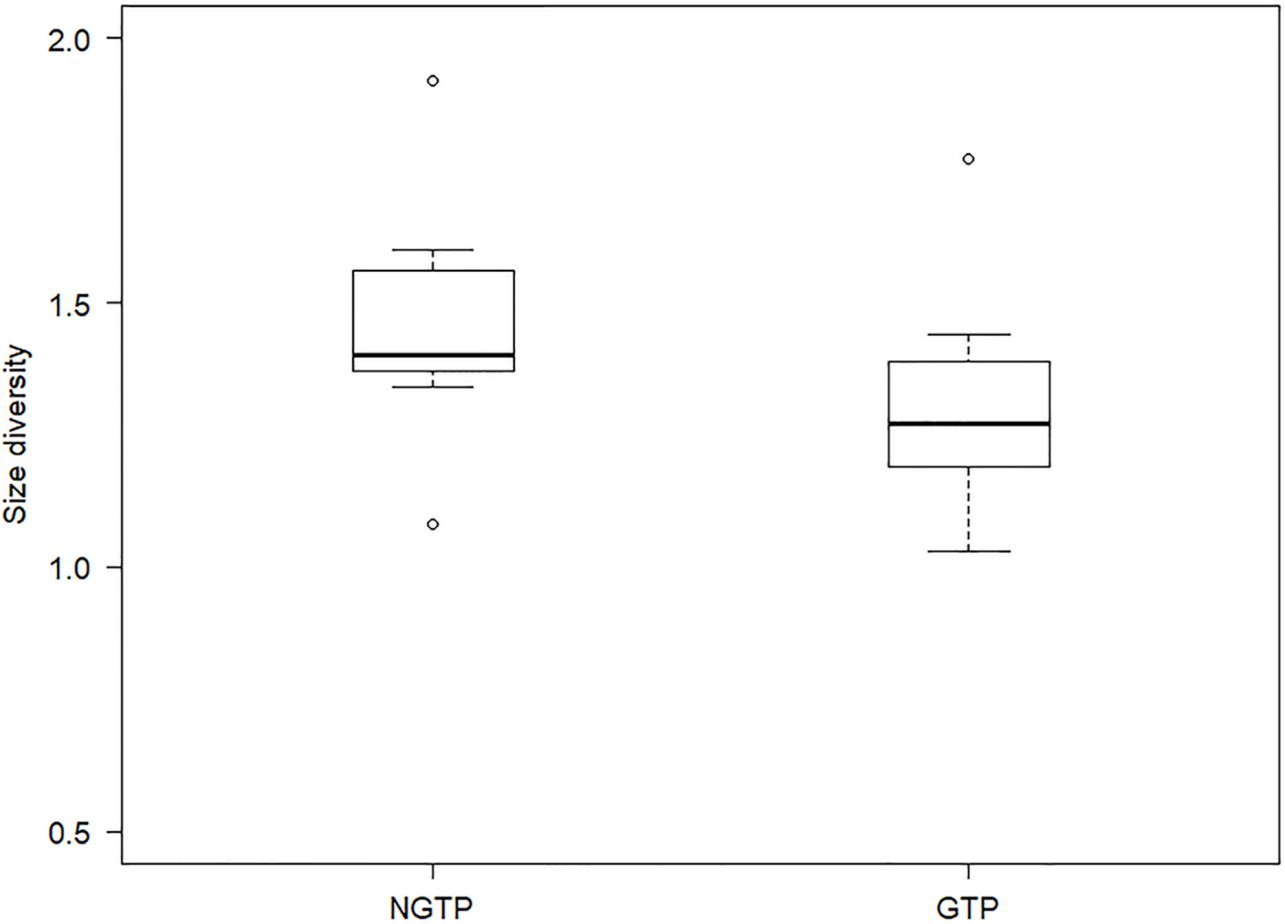
Boxplot of summer zooplankton size diversity during the non-green tide period (NGTP) and green tide period (GTP) at the sampling station. Data for each period were pooled from time series sampling from summer (June–August) of 2005 to 2013. The lower whisker, lower hinge, horizontal line, upper hinge, and upper whisker show minimum, lower quartile, median, upper quartile, and maximum size diversity, respectively.

To determine, in more detail, the effect of GTP vs NGTP on zooplankton size structure, the data were pooled and then separated between the periods. Size diversity (*H′*) was significantly (*P* < 0.05) different between the two periods (Fig. 7). The difference in abundance of each size class between the two periods was shown in Table 3. The results indicated that the abundances of smaller size classes (e.g., 0.5–1.0 mm) significantly (*P* < 0.05) decreased during the GTP, whereas the abundances of other size classes were not significantly changed.

**Table 3:** Comparison of zooplankton abundance in relation to the size classes between the non-green tide period (NGTP) and green tide period (GTP) during summer (June–August) at the sampling station.

Variables	NGTP	GTP	*P*-value
Abundance (ind. m^−3^)			
0.3–0.5 mm	5,610.7 ± 1,367.8	2,265.4 ± 384.0	0.059
0.5–1.0 mm	2,924.7 ± 881.5	658.6 ± 122.7	**0.035**
1.0–2.0 mm	193.4 ± 67.7	91.4 ± 42.5	0.191
2.0–4.0 mm	36.0 ± 15.4	12.7 ± 5.7	0.183
>4.0 mm	15.3 ± 10.8	16.0 ± 9.7	0.964

**Note:**

Differences in abundance of size class between the two defined periods were tested by Wilcoxon test. Abundance values are average ± SE. Bold value corresponds to significant *P*-value at 0.05.

**Figure 8:**
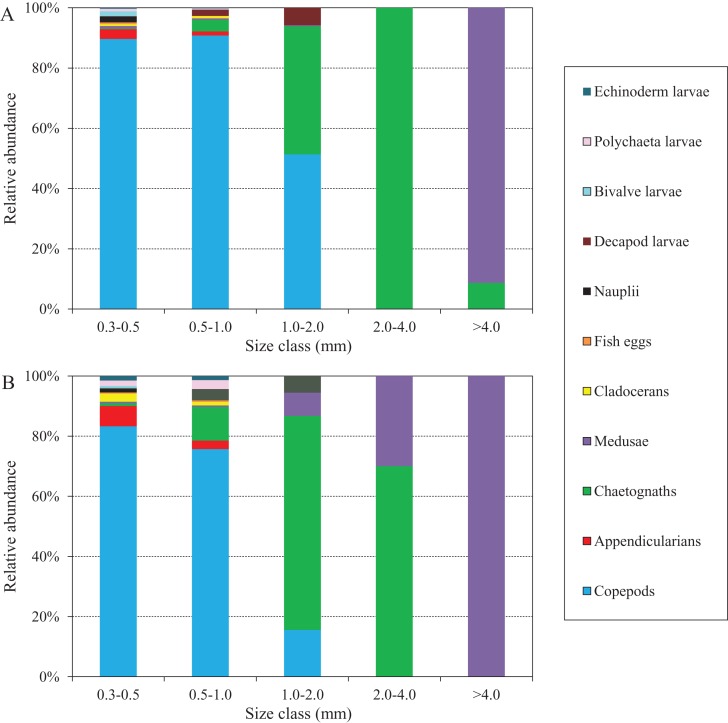
Abundance composition of zooplankton taxonomic groups in each size classes during summer (June–August) at the sampling station. (A) The non-green tide period (NGTP) and (B) green tide period (GTP).

The abundance compositions of zooplankton taxonomic groups in each size class during the two defined periods are shown in Fig. 8, and differed according to the periods. During the NGTP, copepods dominated mainly in the 0.3–0.5, 0.5–1.0, and 1.0–2.0 mm ESD size classes, whereas carnivorous gelatinous zooplankton (e.g., medusae and chaetognaths) were more dominant in the larger size classes (e.g., 2.0–4.0 and >4.0 mm) (Fig. 8A). During the GTP, copepods were dominant only in the 0.3–0.5 and 0.5–1.0 mm ESD size classes, whereas gelatinous zooplankton (including medusae, chaetognaths, and appendicularians) increased their dominance in the 0.5–1.0 and 1.0–2.0 mm ESD size classes (Fig. 8B). It is worth noting that medusae increased in the larger size classes (e.g., 1.0–2.0, 2.0–4.0, and >4.0 mm). In addition, appendicularians, polychaeta larvae, and echinoderm larvae increased their dominance in the smaller size classes (e.g., 0.3–0.5 and 0.5–1.0 mm).

### The relationship between zooplankton abundance and environmental variables

The stepwise GLM model selected allowed a decrease in the AIC from 463.48 to 453.66 (Table 4). The selected GLM model had the following structure: zooplankton ~ SST + SSS + EASMI + AirT + GT, and was statistically significant (*P* = 0.01). The results of the GLM analysis showed that zooplankton abundance in the summers of 2005–2013 at the sampling station depended mainly on sea surface temperature, salinity, EASMI, AirT, and green tide. Specifically, the green tide had a highly significant (*P* = 0.001) effect on the summer zooplankton abundance (Table 4).

**Table 4:** General linear regression model analyses (GLMs).

Combination	AIC
Zooplankton ~ SST + SSS + EASMI + AirT + Chla + PDO + AO + WS + AtmP + Rain + GT	463.48
Zooplankton ~ SST + SSS + EASMI + AirT + Chla + PDO + AO + WS + AtmP + GT	461.49
Zooplankton ~ SST + SSS + EASMI + AirT + Chla + PDO + AO + WS + GT	459.49
Zooplankton ~ SST + SSS + EASMI + AirT + PDO + AO + WS + GT	457.63
Zooplankton ~ SST + SSS + EASMI + AirT + PDO + AO + GT	455.81
Zooplankton ~ SST + SSS + EASMI + AirT + PDO + GT	454.13
Zooplankton ~ SST + SSS + EASMI + AirT + GT	453.66

Source	Type III sum of squares	Mean squares	*F*	*P*-value	*r*^2^
Model	3.52 × 10^8^	7.03 × 10^7^	4.11	**0.010**	0.383
Intercept	1.22 × 10^7^	1.22 × 10^7^	0.71	0.409	
SST	5.25 × 10^7^	5.25 × 10^7^	3.06	0.096	
SSS	2.67 × 10^7^	2.67 × 10^7^	1.56	0.227	
EASMI	4.15 × 10^7^	4.15 × 10^7^	2.42	0.135	
AirT	5.43 × 10^7^	5.43 × 10^7^	3.17	0.090	
GT	2.45 × 10^8^	2.45 × 10^8^	14.28	**0.001**	
Error	3.43 × 10^8^	1.71 × 10^7^			

**Notes:**

Model accounting for the observed variation in zooplankton abundance during summers (June–August) of 2005–2013 at the sampling station according to the results of the GLMs with stepwise selection of variables. Bold values correspond to significant *P*-values at 0.05.

SST, sea surface temperature; SSS, sea surface salinity; EASMI, East Asian Summer Monsoon Index; AirT, air temperature; Chl *a*, chlorophyll *a*; PDO, Pacific Decadal Oscillation; AO, Arctic Oscillation; WS, wind speed; AtmP, atmospheric pressure; GT, green tide.

## Discussion

### Zooplankton community variability

Since the first large-scale occurrence in 2008, *U. prolifera* blooms have occurred in 10 consecutive years and have become an annual summer event in the western Yellow Sea, resulting in serious damage to the environment and ecosystem (Hu, Hu & He, 2017; Zhang, Huo & He, 2017). The present study focuses on the temporal variability in the zooplankton community during these annual macro-algal blooms in the northwestern Yellow Sea shelf. During the 9-year sampling period, this research revealed that the presence of green tides was thought to influence both zooplankton abundance and community structure (e.g., Table 1; Fig. 3). These findings are consistent with previous studies which reported the negative effects of macro-algal blooms on coastal ecosystems (Wang et al., 2011; Lyons et al., 2014; Le Luherne et al., 2017). Overall, our analyses identified evident changes in zooplankton community that may be attributed to the annual green tides, and provided a preliminary assessment of the effects of the green tides on the structure and function of the ecosystem in this region.

One of the major challenges in assessing the impact of green tides on the western Yellow Sea ecosystem is determining the variability in response to the *U. prolifera* blooms to that due to natural environmental “noise.” Zooplankton abundance and assemblage structure are often considered to be affected by a variety of oceanographic conditions, among which water temperature and salinity are the most important factors in zooplankton community structure (Sabates, Gili & Pages, 1989). In addition, large-scale climatic indices (e.g., PDO, AO, and EASMI) and local weather conditions (e.g., wind speed and rain) have been reported to affect the zooplankton community (Hooff & Peterson, 2006; Tseng et al., 2008; Chiba et al., 2015). According to the PCA analysis, the environmental variables in this study are not evidently different between the NGTP and GTP (Fig. 2). Additionally, the GLM results revealed that the interannual variability in zooplankton abundance was significant (*P* = 0.001) correlated with green tides rather than the abovementioned environmental factors (Table 4). Although there may be other factors involved in shaping the community not examined in our study (e.g., abundance of zooplanktivores), we postulate that our observed temporal variations in zooplankton community were possible links to the large-scale *U. prolifera* blooms, having eliminated many other likely factors.

Our study suggests that many zooplankton taxa were present significantly lower abundances during the GTP relative to NGTP (Table 2; Fig. 4), a result that agrees with the previous reports of negative impact of green tides imposed on marine organisms, such as copepods (Franz & Friedman, 2002) and fish (Pihl, Modin & Wennhage, 2005; Le Luherne et al., 2017). When explaining the observed changes in the community structure, both bottom-up and top-down effects should be considered, as they can influence trophic structure (Finlay et al., 2007; Llope et al., 2012). Although food resources for zooplankton are represented by a variety of autotrophic and heterotrophic components, it has been assumed that phytoplankton biomass (Chl *a*) is the key indicator of food conditions (Bachiller & Irigoien, 2015). One possible explanation is that the zooplankton population decreased in response to declined primary productivity (i.e., bottom-up control). In fact, the occurrence of macro-algal blooms has been proved to change the abundance and community structure of phytoplankton (Zhang et al., 2013). For instance, Xing et al. (2015a, 2015b) used MODIS Chl *a* concentration algorithms to analyze the relationship between *U. prolifera* and phytoplankton in the Yellow Sea, and revealed that the macroalgal bloom could result in a significant reduction of phytoplankton biomass. Based on satellite measurements, similarly, Sun et al. (2018) proposed that there was a negative correlation between the occurrence of *U. prolifera* and Chl *a* concentration in the Southern Yellow Sea, specifically, the Chl *a* decreased with the dramatically increased coverage of *U. prolifera* in June, and slowly recovered and finally stabilized as *U. prolifera* decreased in July and August. In this study, the similar temporal variation of Chl *a* has been observed with our field measurement data (Material S1), which confirmed the negative relationship between the green tide and Chl *a*. The laboratory study found that the reproduction of *U. prolifera* was faster than that of the other algae and could inhibit the production and growth of planktonic microalgae by allelochemical secretion and nutritional competition (Liu et al., 2018). During the outbreak stage in June, the *U. prolifera* drifted to the open sea of the southern Shandong Peninsula, including the coastal waters of Qingdao, and there were huge amounts of floating green algae, which required large amount of nutrients to support such a high biomass. But the nutrient in these waters was insufficient, so the *U. prolifera* showed strong inhibitory effect on planktonic microalgae under the coexistence of nutrient competition and allelopathy which lead to the sharp decrease of Chl *a* (Sun et al., 2018). Analogously, Xing et al. (2015a) pointed out that the significant decreased water-column phytoplankton biomass in June was because of the nutrients and light’s consumption by the rapidly increased macroalgae biomass. On the contrary, the Chl *a* rebounded after the collapse of the green tide in late July, since the decomposition of *U. prolifera* may release considerable amounts of ammonium and phosphate into the surrounding seawater to support the recovery growth of phytoplankton (Wang, Yu & Zhou, 2012). Due to the seasonal cycle, food conditions may influence zooplankton dynamics and structure (Vargas, Escribano & Poulet, 2006). This can possible explain the observed evident zooplankton community variability in June rather than in July and August in this study (Table 1).

A second possible explanation for decreased zooplankton abundances is the potential top-down control of the zooplankton community. The pelagic community includes important predators such as jellyfish, chaetognaths, fish larvae, and fish. In this study, we found a positive linear correlation between carnivorous gelatinous zooplankton (i.e., medusae and chaetognaths) and copepods (*r*^2^ = 0.25), suggesting co-occurrence, although not necessarily a top-down control (Gerritsen & Strickler, 1977). Top-down controls may not act individually (Verheye et al., 1992), and the complexity of prey-predator interactions within the zooplankton community are not easily discernible (Bachiller & Irigoien, 2015). In addition, important changes in the structure of pelagic communities can result from a combination of top-down and bottom-up controls (Schmoker & Hernández-León, 2013), and their relative strength may vary across different ecosystems and time (Jeppesen et al., 2003).

Size structure is an important attribute of the community at lower trophic levels (Seguin et al., 2014), and size diversity is often considered one of the major metrics used to measure community size structure (Brucet et al., 2010; Yvon-Durocher et al., 2011), and more useful for indicating variation in the functional structure of the planktonic community (Ke et al., 2018). In the present study, the difference in size structure between the NGTP and GTP revealed that important changes were taking place in the zooplankton community in response to the occurrence of macro-algal blooms (Table 3; Fig. 7). From the perspective of species composition in different size groups, the ratio of copepods decreased in the small size groups (i.e., 0.3–0.5 and 0.5–1.0 mm) and that of gelatinous zooplankton increased, particularly jellyfish in the large size groups, that is, 2.0–4.0 and >4.0 mm (Fig. 8). It is possible that the compositions of other groups were also modified and thus affected the size structure of the whole community.

Changes in the community structure of zooplankton and their links to large-scale macro-algal blooms should be considered as critical in ecosystem functioning in the western Yellow Sea. Species composition modulates trophic interactions, community metabolism, and the energy and carbon fluxes through the food web, with consequences for the productivity of higher trophic levels. The above-mentioned changes in zooplankton composition are related to variations in species and species assemblages. For example, the proportion of dominant species changed as demonstrated by the presence of steeper slopes with more small-sized organisms (Sprules, Munawar & Jin, 1988), and flatter slopes with more diverse taxa and sizes in the community (Pino-Pinuer et al., 2014). A similar situation was observed in this study, as small organisms were predominant during the NGTP due to a greater abundance of copepods (mainly copepods but also other crustaceans) and larval stages (Table 2; Fig. 5), which spawn and reproduce more rapidly under the favorable conditions in spring-summer in this region (Sun et al., 2011a, 2011b). All of the above-mentioned changes can have strong effects on the food chains and C flux, with negative consequences for the carbon pump and commercially important fish populations in this region, such as anchovies (Jin, Zhang & Xue, 2010).

### The potential threats of green tides to the marine ecosystem

Macro-algal blooms are increasing worldwide, and have a number of detrimental effects on marine and estuarine ecosystems (Lyons et al., 2014). The negative ecological effects of macro-algal blooms occur due to alterations in environmental conditions such as the depletion of oxygen in the water column during decomposition and night time respiration and the release of toxic hydrogen sulfide during the decaying process. Thus, these blooms often reduce abundance and alter the biological composition and ecological processes in the ecosystems they affect (Raffaelli, Raven & Poole, 1998). During the *U. prolifera* bloom studied herein, we found that both the abundance and relative abundance of copepods decreased, and the relative abundance of gelatinous zooplankton (e.g., jellyfish) and some meroplankton increased (Figs. 5 and 8).

Copepods are dominant in the mesozooplankton in oceans and play a vital role in transferring primary production to higher trophic levels (e.g., fish larvae and fish) in all aquatic ecosystems (Verheye et al., 1992). Changes in the abundance of copepods have an important impact on the dynamics of fishery resources and the main food web. For instance, a reduction in the abundance of *Acartia tonsa* contributed to a decline in the zooplanktivorous bay anchovy (*Anchoa mitchilli*) and menhaden (*Brevoortia tyrannus*) in the Maryland portion of Chesapeake Bay (Kimmel, Boynton & Roman, 2012). In situ research confirmed that large-scale harmful algal blooms could decrease the abundance of copepods, and simultaneously increase the abundance and dominance of jellyfish (Lin et al., 2014). In this study, we found that the relative abundance of jellyfish increased during the GTP; therefore, we suggest that these large-scale macro-algal blooms probably promote the widespread occurrence of jellyfish in the context of global change. Over the past several decades, jellyfish populations have exhibited an increasing trend in estuarine and coastal ecosystems around the world (Mills, 2001; Brotz et al., 2012; Condon et al., 2012). The massive occurrence of carnivorous zooplankton often pose a major threat to fishery resources, either through direct predation on larval stages, or through competition for zooplankton prey (Mutlu, 2001; Brodeur, Sugisaki & Hunt, 2002). It is believed that frequent jellyfish blooms can be a threat to the sustainability of fisheries in the East Asian Marginal Sea, one of the world’s most productive fishing grounds (Uye, 2008). The decrease both in abundance and proportion of copepods that accompanies the increased relative abundance of jellyfish may pose a risk to fish recruitment and, ultimately, result in the depletion of fishery resources.

To date, many studies have indicated that macro-algal blooms can have a significant impact on zoobenthic communities, such as a decrease in the species richness of zoobenthos (Norkko & Bonsdorff, 1996b), an increase in the number of opportunistic species (Thrush, 1986; Bolam et al., 2000), and the migration and mass mortality of some species (Norkko & Bonsdorff, 1996a). Wang, Yu & Zhou (2011) reported that large-scale macro-algal blooms had a negative impact on the benthic community in the western Yellow Sea. Wu et al. (2010) demonstrated that the biomass of meiofauna in summer 2008 decreased by approximately 1/3 compared to summer 2007 in the Yellow Sea. Moreover, Hua et al. (2015) suggested that the abundance of meiofauna decreased and polychaeta increased and dominated the meiobenthos community following the *U. prolifera* bloom in the summer of 2012. It is obvious that large-scale *U. prolifera* blooms have negative effects on the benthic community, and thus influence planktonic larval recruitment and change the meroplankton community structure. In this study, we found that the dominant species of meroplankton completely changed during the GTP (Fig. 6). Specifically, polychaeta larvae and echinoderm larvae were dominant over decapod larvae and bivalve larvae, and dominated the meroplankton. The increased abundance and dominance of echinoderm larvae in the summer plankton represents a major change in the balance between the meroplankton and the holoplankton, and is indicative of a shift in resource partitioning between the benthos and pelagos (Kirby et al., 2007). As a result, the above changes in the benthic community may alter the trophodynamics of the summer pelagic ecosystem through competition between its larvae and holozooplankton taxa, and have a significant impact higher up the food chain in the western Yellow Sea.

In the past decade, large-scale *U. prolifera* blooms have taken place in the western coastal Yellow Sea each year, affecting areas as large as 10^4^ km^2^. As macro-algal blooms are predicted to intensify with rising temperatures and increased eutrophication (Gao et al., 2017), this may lead to the accumulation of these negative effects and, ultimately, result in degradation of the marine ecosystem. Thus, the ecological impacts associated with these changes need to be continuously monitored in order to preserve these fragile ecosystems.

## Conclusion

Our results indicate a significant temporal variability in the zooplankton community and its possible links to green tides in our study region off Qingdao coast, in the western Yellow Sea. A significant decrease in zooplankton abundance (mainly copepods) was observed during the outbreak stage in June, chiefly attributed to the decreased primary productivity. As most planktivorous fish are visual predators and select prey based on both type and size, the temporal changes in zooplankton community could disrupt fisheries by affecting predator-prey dynamics between larval fish and their primary copepod prey. Therefore, the green tide could affect the primary and secondary producers, and influence the energy transfer through the food chains. This study provided fundamental knowledge that identify potential threats posed by large-scale *U. prolifera* blooms to the coastal ecosystem. However, a better understanding of the interactions between zooplankton and the bloom-forming *U. prolifera* is necessary in order to understand the mechanisms and ecological consequences of green tides in the western Yellow Sea.

## Supplemental Information

10.7717/peerj.6641/supp-1 Supplemental Information 1 Monthly variability in mean chlorophyll *a* (mg L^−1^) during the non-green tide period (NGTP) and green tide period (GTP) at the sampling station. Vertical bars show SE.

10.7717/peerj.6641/supp-2 Supplemental Information 2 Biological and environmental raw data.

## Additional Information and Declarations

### Data Availability

The following information was supplied regarding data availability: The raw measurements are available in Files S1 and S2.
