# Supplementary figures and images for: Temporal variability in zooplankton community in the western Yellow Sea and its possible links to green tides
Source: PeerJ. 2019 Apr 8;7:e6641. doi: 10.7717/peerj.6641 (PMC6459317; doi:10.7717/peerj.6641)

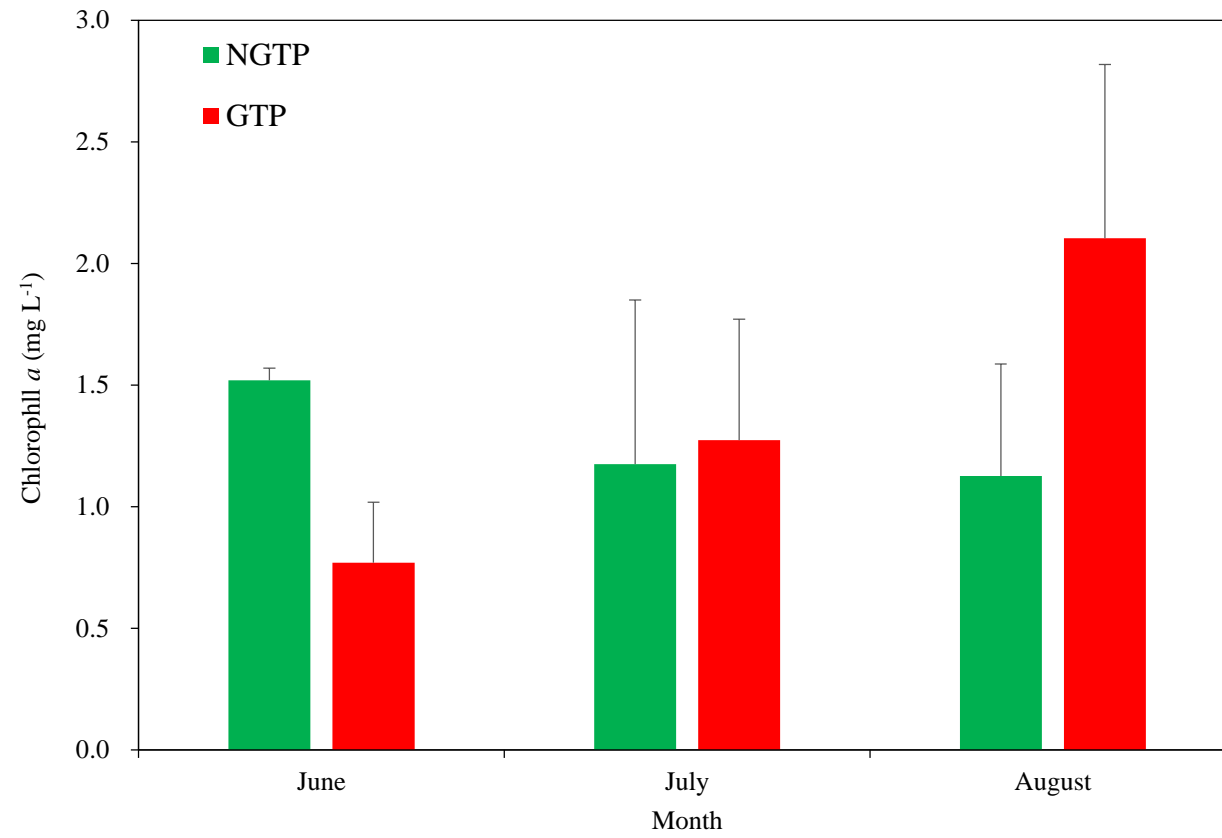

Supplement: Supplemental Information 1 — Vertical bars show SE. [file peerj-07-6641-s001.pdf]
